# Supplementary material for: The interactome of intact mitochondria by cross-linking mass spectrometry provides evidence for coexisting respiratory supercomplexes
Source: Mol Cell Proteomics. 2017 Dec 8;17(2):216–32. doi: 10.1074/mcp.RA117.000470 (PMC5795388; doi:10.1074/mcp.RA117.000470)
Supplement: Supplemental Data [file supp_RA117.000470_133922_0_supp_23978_fzffwf.zip › spectra_annotation/mito_spectra_annotation/239-1-5-1-5-1.pdf]

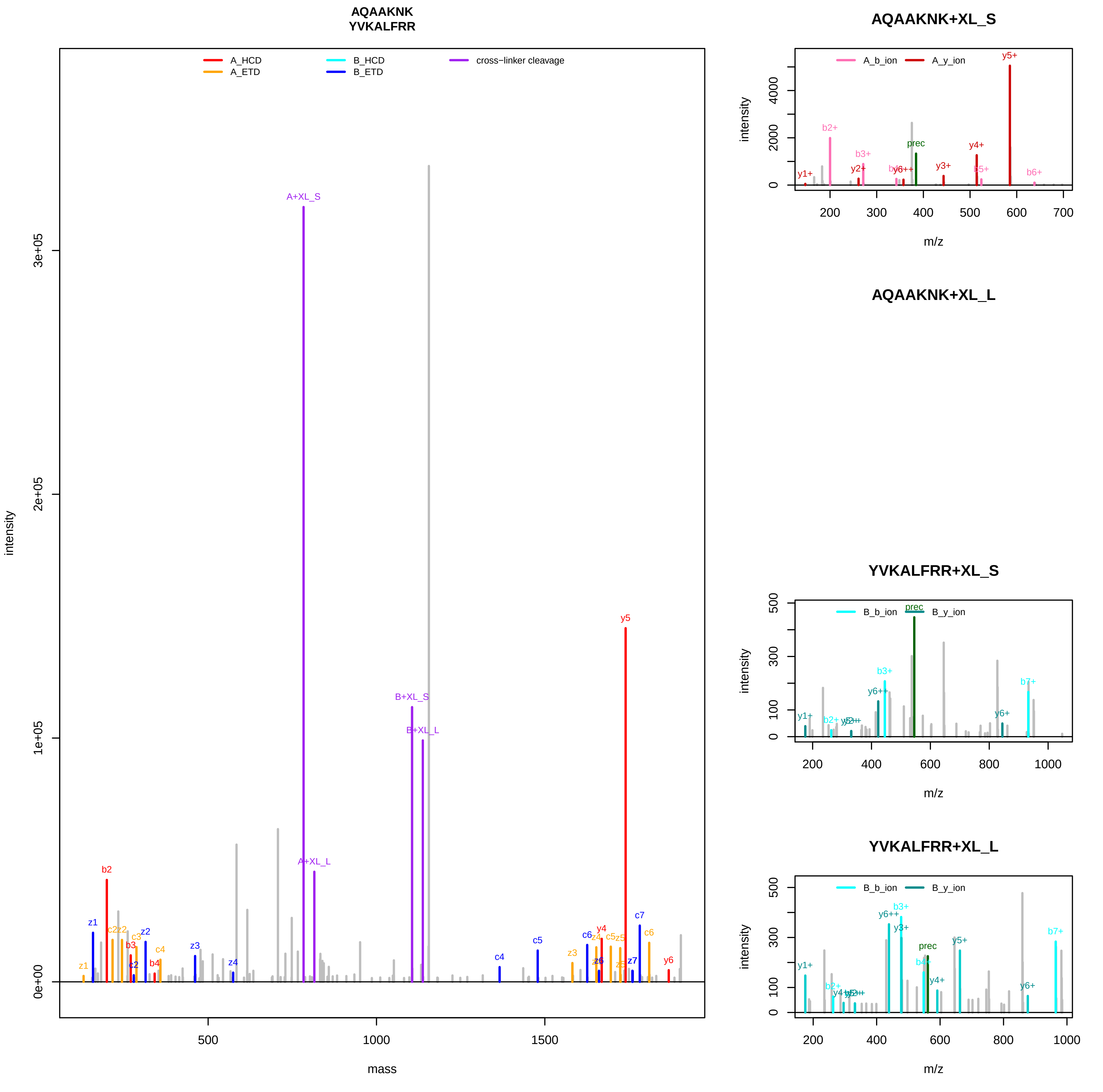
AQAAKNK
YVKALFRR
A_HCD
A_ETD
B_HCD
B_ETD
cross−linker cleavage
intensity
mass
0e+00
1e+05
2e+05
3e+05
500
1000
1500
A+XL_S
A+XL_L
B+XL_S
B+XL_L
b2
b3
b4
y4
y5
y6
z1
c2
z2
z3
z4
c4
c5
c6
z6
c7
z7
c2z2
c3
z5
c5z5
AQAAKNK+XL_S
A_b_ion
A_y_ion
b2+
b3+
y1+
y2+
y6++
prec
y3+
y4+
b5+
y5+
b6+
m/z
AQAAKNK+XL_L
YVKALFRR+XL_S
B_b_ion
B_y_ion
y1+
b2+
b3+
y6++
y6+
b7+
YVKALFRR+XL_L
y3+
y4+
y5+
b4+
intensity
